# A study of stress, composition and grain interaction gradients in energy-dispersive X-ray stress analysis on materials with cubic symmetry

**DOI:** 10.1107/S1600576724003996

**Published:** 2024-06-07

**Authors:** Christoph Genzel, Manuela Klaus

**Affiliations:** a Abteilung für Mikrostruktur- und Eigenspannungsanalyse Helmholtz-Zentrum Berlin für Materialien und Energie Albert-Einstein-Strasse 15 12489Berlin Germany; Montanuniversität Leoben, Austria

**Keywords:** X-ray stress analysis, energy-dispersive diffraction, grain interaction, composition gradients, stress gradients

## Abstract

The influence of various combinations of residual stress, composition and grain interaction gradients in polycrystalline materials with cubic symmetry on energy-dispersive X-ray stress analysis is theoretically investigated. For the evaluation of the simulated sin^2^ψ distributions, two different strategies are compared with regard to their suitability for separating the individual gradients. It is shown that the separation of depth gradients of the strain-free lattice parameter *a*_0_(*z*) from residual stress gradients σ(*z*) is only possible if the data analysis is carried out in section planes parallel to the surface. The impact of a surface layer z* that is characterized by a direction-dependent grain interaction model in contrast to the volume of the material is quantified by comparing a ferritic and an austenitic steel, which feature different elastic anisotropy. It is shown to be of minor influence on the resulting residual stress depth profiles if the data evaluation is restricted to reflections *hkl* with orientation factors Γ^*hkl*^ close to the model-independent orientation Γ*. Finally, a method is proposed that allows the thickness of the anisotropic surface layer z* to be estimated on the basis of an optimization procedure.

## Abstract 2

The influence of various combinations of residual stress, composition and grain interaction gradients in polycrystalline materials with cubic symmetry on X-ray stress analysis by energy-dispersive diffraction is discussed on the basis of simulations for ferritic and austenitic steel.

## 1. Introduction

X-ray stress analysis (XSA) on polycrystalline materials is based on the experimental determination of lattice strains 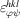 for one or more reflections *hkl* in various directions (φ, ψ) with respect to the sample reference system, from which individual components of the stress tensor are then determined using Hooke’s law (Noyan & Cohen, 1987 ▸; Hauk, 1997 ▸). The required diffraction elastic constants (DECs) usually differ from the mechanical constants (Young’s modulus, shear modulus, compression modulus *etc*.), since the lattice strains determined on the simultaneously diffracting crystallites refer to different crystal directions, which feature an anisotropic elastic behavior. The DECs can be either determined experimentally or calculated from the elastic single-crystal constants using models that describe the elastic interaction between the grains on the basis of various assumptions.

For materials with random crystallographic and morphological texture, the grain interaction models range between the limiting assumptions of homogeneous strain (Voigt, 1910 ▸) and homogeneous stress (Reuss, 1929 ▸) in all crystallites. Fre­quently used approaches go back to Eshelby–Kröner [elastic polarizability of the crystallites (Eshelby, 1957 ▸; Kröner, 1958 ▸)] and Hill/Neerfeld [arithmetic mean of Reuss and Voigt (Neerfeld, 1942 ▸; Hill, 1952 ▸)]. The DECs obtained with these models and usually denoted by 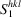 and 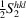 are characterized by their different behavior on different length scales. On the microscopic (grain) scale they depend (except the Voigt model) on the measured reflection *hkl* and, therefore, reflect the elastic single-crystal anisotropy. Within the volume probed by the X-ray beam, the DECs do not depend on the measure­ment direction (φ, ψ), thus reflecting the (quasi-)isotropic material behavior on the macroscopic scale.

Materials with pronounced crystallographic texture are elastically anisotropic on the macroscopic scale as well and therefore require a different treatment in the context of XSA. In order to link the measured lattice strains 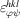 in these cases in a linear way with the components σ_*ij*_ of the macroscopic stress tensor, the concept of stress factors *F_ij_*[*hkl*, φ, ψ, *f*(*g*)] was introduced by Dölle & Hauk (1978 ▸, 1979*b* ▸), where the function *f*(*g*) takes into account the orientation distribution of the crystallites in the material [see also Brakman (1987 ▸)]. In various publications, the stress factor approach was used to determine residual stresses (Marciszko-Wiackowska *et al.*, 2019 ▸) and their depth distribution (Klaus & Genzel, 2017 ▸; Klaus *et al.*, 2017 ▸; Marciszko *et al.*, 2018 ▸) separately from the strain-free lattice parameter by means of multi-reflection methods performed in the angle-dispersive (AD) and energy-dispersive (ED) diffraction mode, respectively.

Macroscopic anisotropy in polycrystalline materials can occur even in the absence of crystallographic texture. This phenomenon has been observed in (very) thin films experimentally (Kumar *et al.*, 2006 ▸; Welzel *et al.*, 2002 ▸, 2009 ▸) and was studied theoretically (van Leeuwen *et al.*, 1999 ▸; Kamminga *et al.*, 2000 ▸; Leoni *et al.*, 2001 ▸; Koch *et al.*, 2004 ▸) [see also Welzel *et al.* (2003 ▸) and Welzel & Mittemeijer (2003 ▸)] on the basis of the direction-dependent grain interaction model proposed by Vook and Witt (Vook & Witt, 1965 ▸; Witt & Vook, 1968 ▸). The reason for this material behavior is considered to be that the crystallites in thin layers are only surrounded by other crystallites in two dimensions, which can result in an (extremely) unconstrained expansion in the film normal direction (Reuss case) and a (completely) constrained, *i.e.* identical, deformation of all crystallites in the film plane (Voigt case).

An extension of the concept of anisotropic grain interaction to the near-surface region of bulk materials was introduced by Baczmanski *et al.* (2003 ▸, 2008 ▸). The ‘self-consistent free-surface model’ is based on the assumption that the crystallites in the uppermost surface layer behave elastically, similar to the model proposed by Vook & Witt (1965 ▸). Within this layer this model supposes free expansion according to Reuss in the out-of-plane direction and partially constrained in-plane deformation according to Eshelby–Kröner. In the material volume below a quasi-isotropic elastic behavior is assumed, which is described by the Eshelby–Kröner model. Crystallographic texture is taken into account by means of the orientation distribution function *f*(*g*).

**Figure 1:**
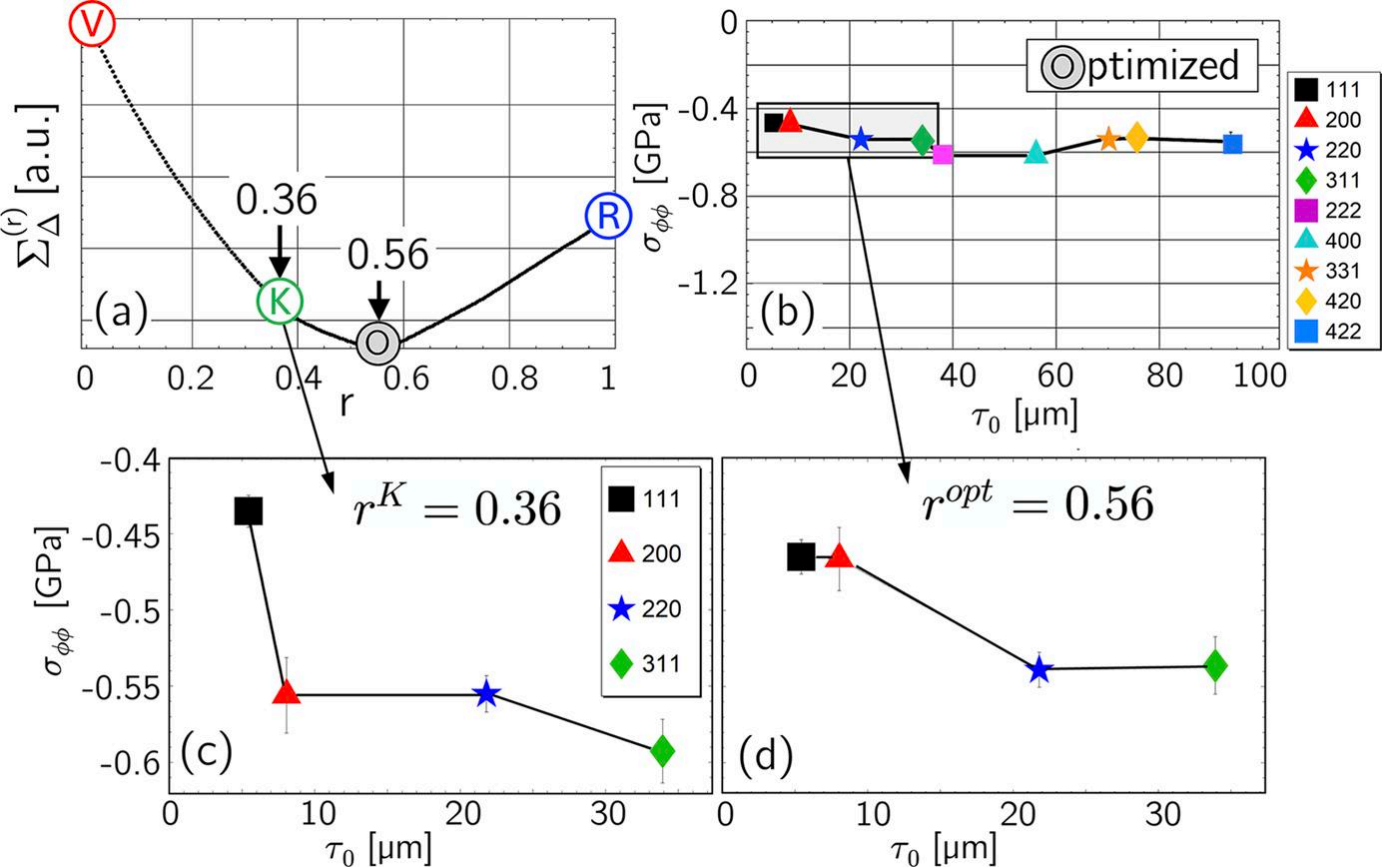
Optimization procedure for refining the residual stress depth profile and the grain interaction model in the near-surface region of an austenitic stainless steel of type TP347H (ASTM A213), which is commercially applied for superheaters in thermal power plants. Example taken from Genzel *et al.* (2023 ▸). (*a*) Minimization of the total path length between the individual stress values shown in diagram (*b*). The diagrams (*c*) [stress depth profile determined for the Eshelby–Kröner (K) grain interaction model] and (*d*) [depth profile obtained for the optimized (O) grain interaction model] represent section enlargements of the area closest to the surface. See text for further details.

Experimental studies indicate that the Eshelby–Kröner model does not always fully describe the grain interaction in bulk materials. Thus, the example in Fig. 1 ▸ reveals that the formalism proposed by Klaus & Genzel (2019 ▸) for determining the grain interaction model only leads to a smooth profile of the discrete residual stress depth distribution without large jumps between neighboring data points [here between 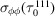 and 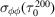 in diagram (*c*)] if the weighting factor *r*, which denotes the Reuss part in the model [see equations (16*a*[Disp-formula fd16a]), (16*b*[Disp-formula fd16b]) and Fig. 18 in Appendix *A*[App appa]], is different from that valid for the Eshelby–Kröner model. The analysis was performed using high-energy ED diffraction [the details are given by Genzel *et al.* (2023 ▸)]. This finding suggests that grain interaction in the topmost surface layer may be different from that in the volume below, emphasizing the need for further investigation of depth-dependent grain interaction.

Very recently a modification of the free-surface model was suggested by Marciszko-Wiackowska *et al.* (2022 ▸). The ‘tunable free-surface model’ explicitly considers the depth dependency of the grain interaction by introducing two weight functions which describe the variation of grain interaction with depth below the surface. In the same publication the new approach was experimentally verified and applied to the non-destructive and depth-resolved analysis of the residual stress state in a mechanically polished austenitic stainless steel specimen in order to simultaneously determine depth profiles of the in-plane residual stresses and grain interaction.

**Figure 2:**
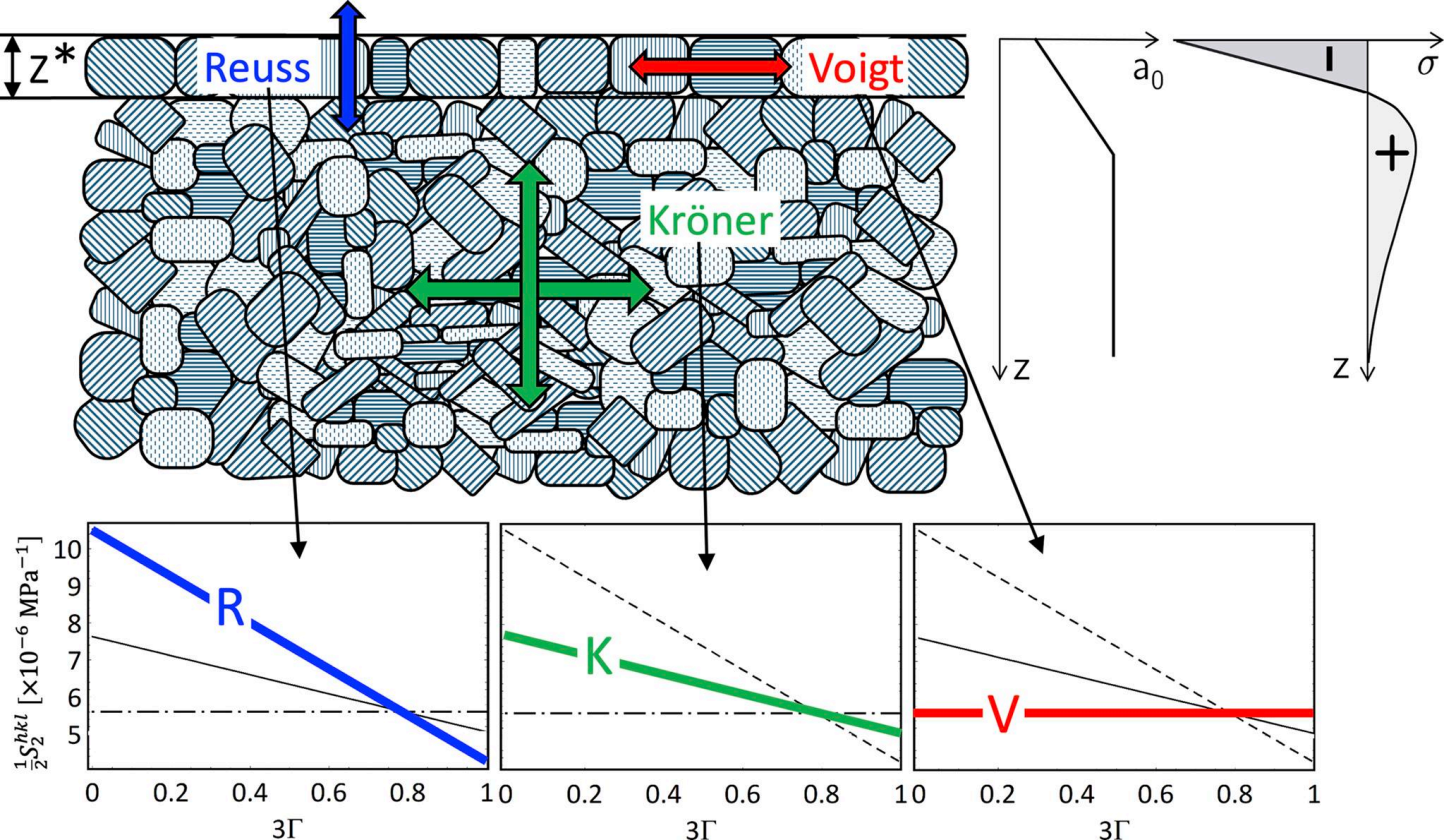
Schematic of the superposition of depth-dependent grain interaction, residual stress distribution and composition in a polycrystalline material. The diffraction elastic constants 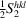 in the diagrams refer to ferritic steel.

The present paper ties in with the considerations of Marciszko-Wiackowska *et al.* (2022 ▸) and raises further questions arising in this context (see Fig. 2 ▸). We assume a polycrystalline material with random crystallographic and morphological (shape) texture, whose near-surface layer with a thickness 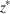 should behave as elastically anisotropic in the sense of the model proposed by Vook & Witt (1965 ▸), while the bulk should be characterized by a quasi-isotropic material behavior according to the Eshelby–Kröner model. Note that the term anisotropy in the present work refers exclusively to the direction-dependent grain interaction, but not to a crystallographic or morphological texture of the crystallites. Furthermore, various combinations of the in-plane residual stress state and the chemical composition (the latter represented, for example, by the carbon content in steel) in the form of σ(*z*) and *a*_0_(*z*) gradients are considered in the surface area covered by the X-ray beam.

For these complex material states, normalized 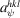
–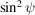 distributions are generated on the basis of simulated ED-XSA experiments, which are then evaluated by means of the modified multi-wavelength plot method (Genzel *et al.*, 2004 ▸) and the constant-τ method (Klaus & Genzel, 2017 ▸). However, as the extension 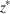 of the anisotropic surface layer is not known in reality, the evaluation starts with the worst-case scenario, in which the 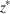 layer is not taken into account. The matrix of investigated scenarios is completed by the application to dif­ferent steel modifications (ferrite and austenite), which differ significantly from each other with regard to elastic single-crystal anisotropy 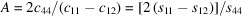 (*c*_*ij*_ and *s*_*ij*_ are the single-crystal elastic constants and moduli, respectively). Finally, it is shown that an optimization procedure introduced by Klaus & Genzel (2019 ▸) to refine the DEC model for the case of homogeneous grain interaction (see Fig. 1 ▸) is also applicable to the depth-dependent case, where it can be used to estimate the depth 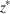. The strategy outlined here allows us to answer the following questions:

(i) How strongly does the grain interaction influence energy-dispersive X-ray stress analysis?

(ii) How can the influence of elastic single-crystal anisotropy be minimized in the data evaluation?

(iii) To what extent does an inhomogeneous chemical composition influence the results obtained with the evaluation methods considered here?

## 2. Modeling direction- and depth-dependent grain interaction

### 2.1. Near-surface and volume stress factors

The following considerations refer to a near-surface material condition as shown in Fig. 2 ▸. The residual stress state, which is assumed to be biaxial and rotationally symmetric (*i.e.* σ_11_ = σ_22_ ≡ σ_∥_, σ_*i*3_ ≡ 0 for *i* = 1, 2, 3), the chemical composition represented by the strain-free lattice parameter *a*_0_ and the grain interaction are supposed to be depth dependent. Using the stress factor concept the fundamental equation of XSA can be written as follows: 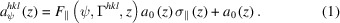
(Γ^*hkl*^ is the orientation factor, see Appendix *A*[App appa].) In the above equation and all following equations 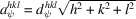 are the lattice spacings normalized to the edge length of the cubic unit cell, *a*^100^. Owing to Beer’s exponential attenuation law for X-rays passing through matter, the experimentally accessible quantities *g* (here *g* can stand for the lattice spacings, stress factors and stresses) are not the actual depth profiles in real space, *g*(*z*), but their Laplace transforms, *g*(τ). The correlation between the *g*(*z*) and the *g*(τ) profiles is given by (Dölle & Hauk, 1979*a* ▸)
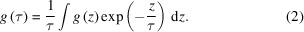
τ is the ‘1/*e* information depth’ valid for thick (bulk) material, where 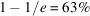 of the total diffracted power *P*^∞^ originates from (Klaus & Genzel, 2013 ▸).

Assuming a macroscopically anisotropic surface layer of thickness 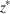 (see Fig. 2 ▸), we define the depth-dependent stress factors as follows: 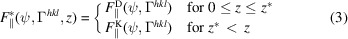
with 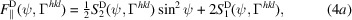

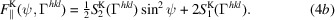
In the above equations D and K stand for the direction-dependent (near-surface) and the Eshelby–Kröner (volume) grain interaction model, respectively (see Appendices *A*[App appa] and *B*[App appb]).

### 2.2. Depth and direction dependence of the stress factors

**Figure 3:**
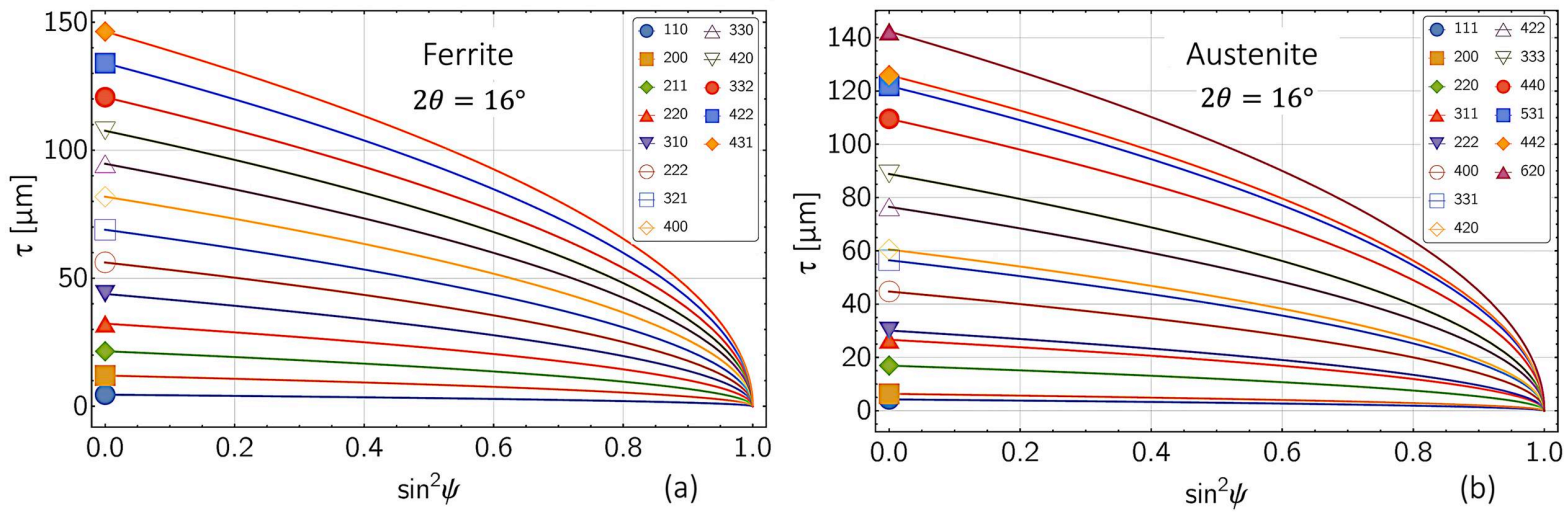
Information depth τ for (*a*) ferritic and (*b*) austenitic steel which would be achieved in ED-XSA measurements performed under a diffraction angle 2θ = 16°.

The Laplace transform of 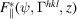 is given by
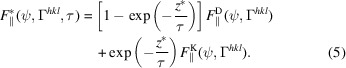
The information depth τ refers to XSA measurements in ED diffraction mode, where each reflection *hkl* has to be assigned to a different photon energy *E*^*hkl*^. For measurements performed in the symmetric Ψ mode, one finds
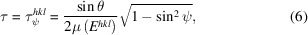
where θ is the Bragg angle, which is fixed during the measurement, and μ denotes the energy-dependent linear absorption coefficient. Fig. 3 ▸ shows the corresponding information depths τ for ferritic and austenitic steel according to equation (6).

**Figure 4:**
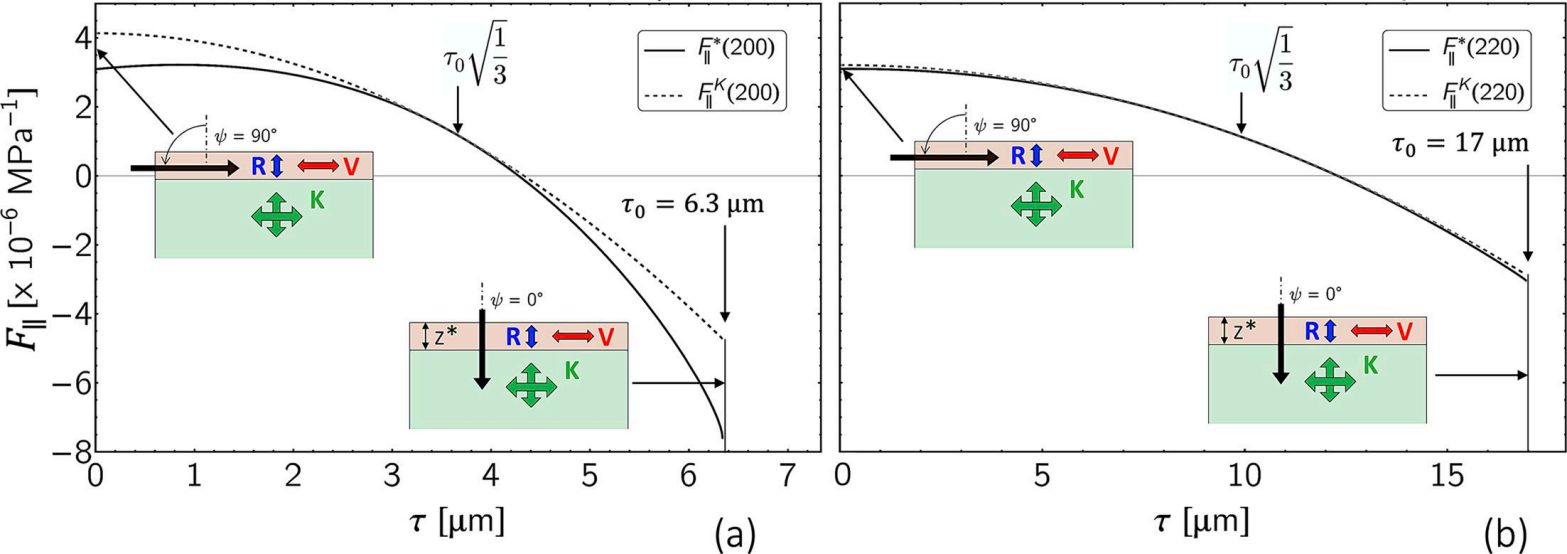
Depth dependence of the stress factor 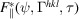 for austenitic steel according to equation (5), calculated for two reflections (*a*) far from and (*b*) close to the model-independent orientation 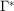. The solid line describes the case 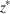 = 10 µm, while the dashed line was calculated for 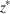 = 0 and thus represents the Eshelby–Kröner (volume) case.

Equation (5) reveals that the stress factors depend on the information depth τ as well as on the measurement direction ψ in the sample reference coordinate system and on the crystal direction *hkl*. The depth dependence of the stress factor 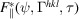 is demonstrated in Fig. 4 ▸. It can be seen that the anisotropic near-surface grain interaction model has a significant influence on the stress factor depth profile if the orientation factor Γ^*hkl*^ of the considered reflection *hkl* is far away from the model-independent orientation 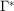 (here the 200 reflection, see also Appendix *A*[App appa]). However, if Γ^*hkl*^ ≃ 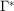 (here the 220 reflection), the depth profile taking into account the surface layer 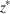 almost coincides with that obtained for the isotropic volume grain interaction model. It is also noticeable that the curves for both grain interaction models touch at a depth of 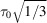, where τ_0_ is the maximum information depth achieved for ψ = 0.

**Figure 5:**
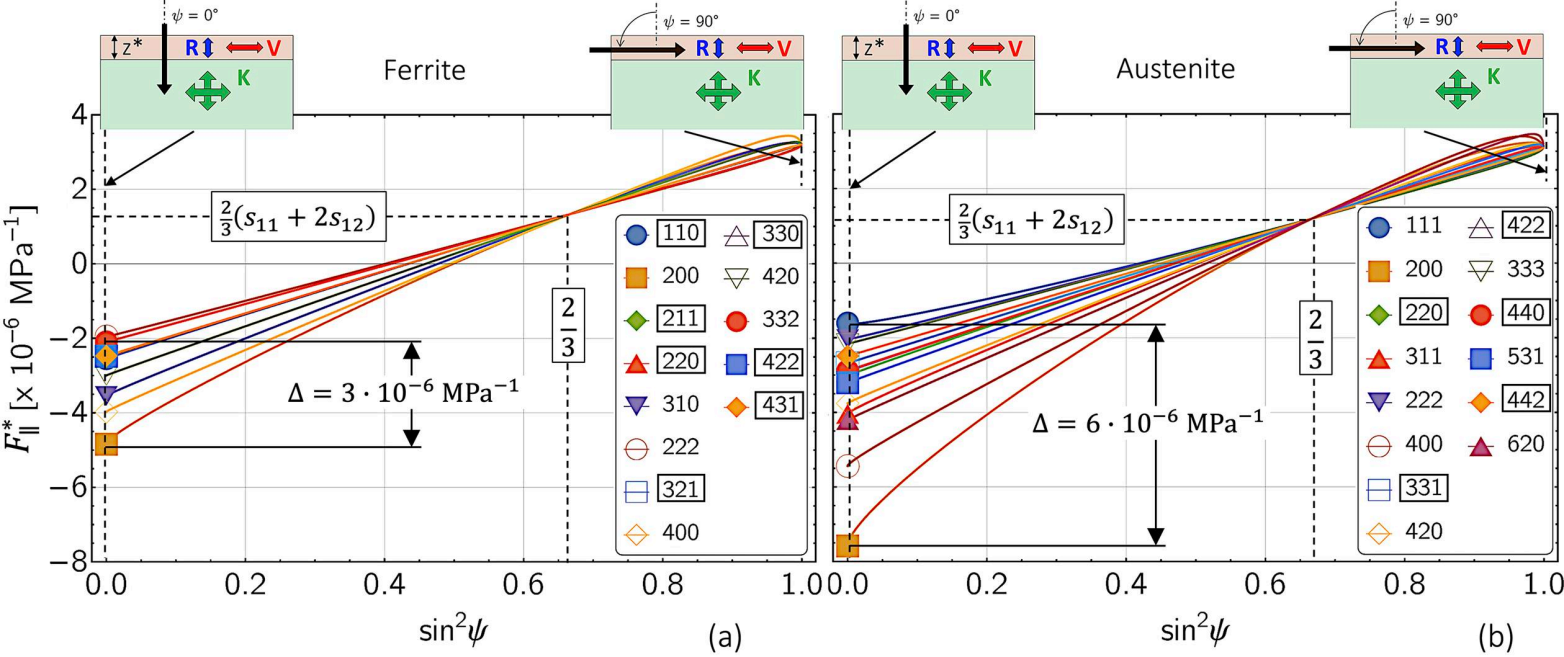
Direction dependence of the stress factor 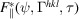 for (*a*) ferritic and (*b*) austenitic steel according to equation (5), calculated for the case 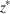 = 10 µm. Δ denotes the difference between the limiting cases 3Γ^*hhh*^ = 1 and 3Γ^*h*00^ = 0. The framed indices *hkl* mark the crystal directions close to the model-independent orientation 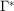 (see Appendix *A*[App appa]).

The reason for the latter finding becomes clear if one considers the directional dependence of the stress factors (see Fig. 5 ▸). There, all stress factors intersect at the same point 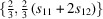 regardless of the orientation factor Γ^*hkl*^. [This result is obtained by equating the stress factors 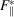 and 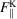 from equations (5) and (4*b*[Disp-formula fd4b]) and expressing the DECs contained therein by the single-crystal moduli *s*_*ij*_ (see Appendix *A*[App appa]).] Since *s*_11_ + 2*s*_12_ = (3*K*)^−1^ applies to cubic materials (*K* is the identical compression modulus for cubic and isotropic materials), the ordinate of the intersection can be regarded as the ‘plane compression modulus’ for the biaxial rotationally symmetrical stress state assumed here. The 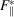 versus 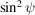 curves further reveal a more linear behavior, the closer the corresponding reflections *hkl* are to the model-independent orientation 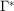. Finally, the stronger anisotropy of austenite becomes apparent from the significantly larger spread of the curves for 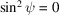, which corresponds to the Reuss case of the direction-dependent surface layer model.

## 3. Data evaluation strategies

The simulation strategy is based on the following assumptions. A depth-dependent grain interaction model as shown in Fig. 2 ▸, consisting of a surface layer of thickness 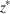 with a direction-dependent DEC model and an isotropic DEC model in the volume below, is assumed for the generation of the ‘measurement data’. For the data analysis, *i.e.* the evaluation of the residual stress and composition depth profiles, the ‘wrong’ volume grain interaction model is used. In this way, the deviations that arise when the (generally unknown) depth-dependent grain interaction model is not taken into account can be quantified and assessed. Finally, this approach allows us to derive recommendations on how to minimize the influence of the grain interaction model on the ED-XSA.

### 3.1. The modified multi-wavelength plot method

**Figure 6:**
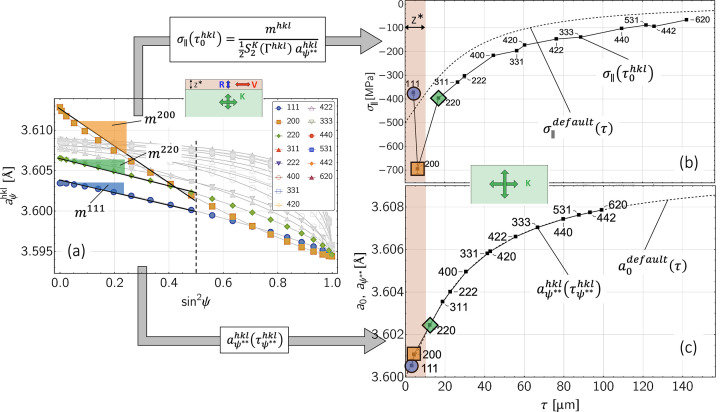
Principle of the modified multi-wavelength method. The different pictograms in this and all following figures are intended to indicate that the actual DEC models (*i.e.* directional in the surface layer, isotropic in volume) are used for data generation, while the volume model is assumed for data analysis (*i.e.* stress evaluation). The simulated sin^2^ψ curves in (*a*) refer to the case of an austenitic steel and a diffraction angle 2θ = 16°. (*b*) and (*c*) Depth profiles obtained for the in-plane stress and the strain-free lattice parameter, respectively. For the surface layer a thickness 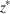 = 10 µm was assumed. See text for further details.

The modified multi-wavelength plot (MMWP) method is an extension of the 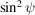 method (Macherauch & Müller, 1961 ▸) to depth-resolved analyses. Originally developed for the AD case of diffraction (Eigenmann *et al.*, 1990 ▸), the approach was transferred to the ED diffraction mode by Genzel *et al.* (2004 ▸) [see also Ruppersberg (1997 ▸)] and later extended to include the possibility of refining a depth-independent DEC model in addition to the stress–depth profiles (Klaus & Genzel, 2019 ▸; Genzel *et al.*, 2023 ▸). The simulation strategy for applying the MMWP approach is depicted in Fig. 6 ▸. The underlying 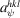
–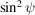 distributions in Fig. 6 ▸(*a*) were generated for the actual (*i.e.* assumed) depth-dependent DEC model by 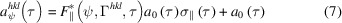
with 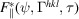 defined by equation (5).

The data analysis (*i.e.* the evaluation of the residual stress and composition depth profiles) is carried out under the assumption that the volume (Eshelby–Kröner) grain interaction model is valid for the entire material: 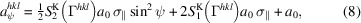
with the DECs 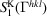 and 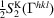 defined by equations (16*a*[Disp-formula fd16a]) and (16*b*[Disp-formula fd16b]) (see Appendix *A*[App appa]). Applying the 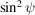 method according to equation (8) to the approximately linear part of the individual 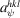
–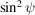 distributions up to 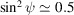 [Fig. 6 ▸(*a*)] provides discrete depth profiles of the residual stress, σ_∥_ [Fig. 6 ▸(*b*)], and the strain-free lattice parameter, 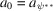 [Fig. 6 ▸(*c*)], if the respective values are assigned to the maximum information depth 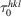 achieved for ψ = 0 (for the stresses) and to the information depth 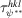 (for the lattice parameter). Tthe latter corresponds to the strain-free direction 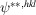 of the biaxial stress state defined by 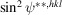 = 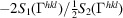.

### 3.2. The constant-τ method

**Figure 7:**
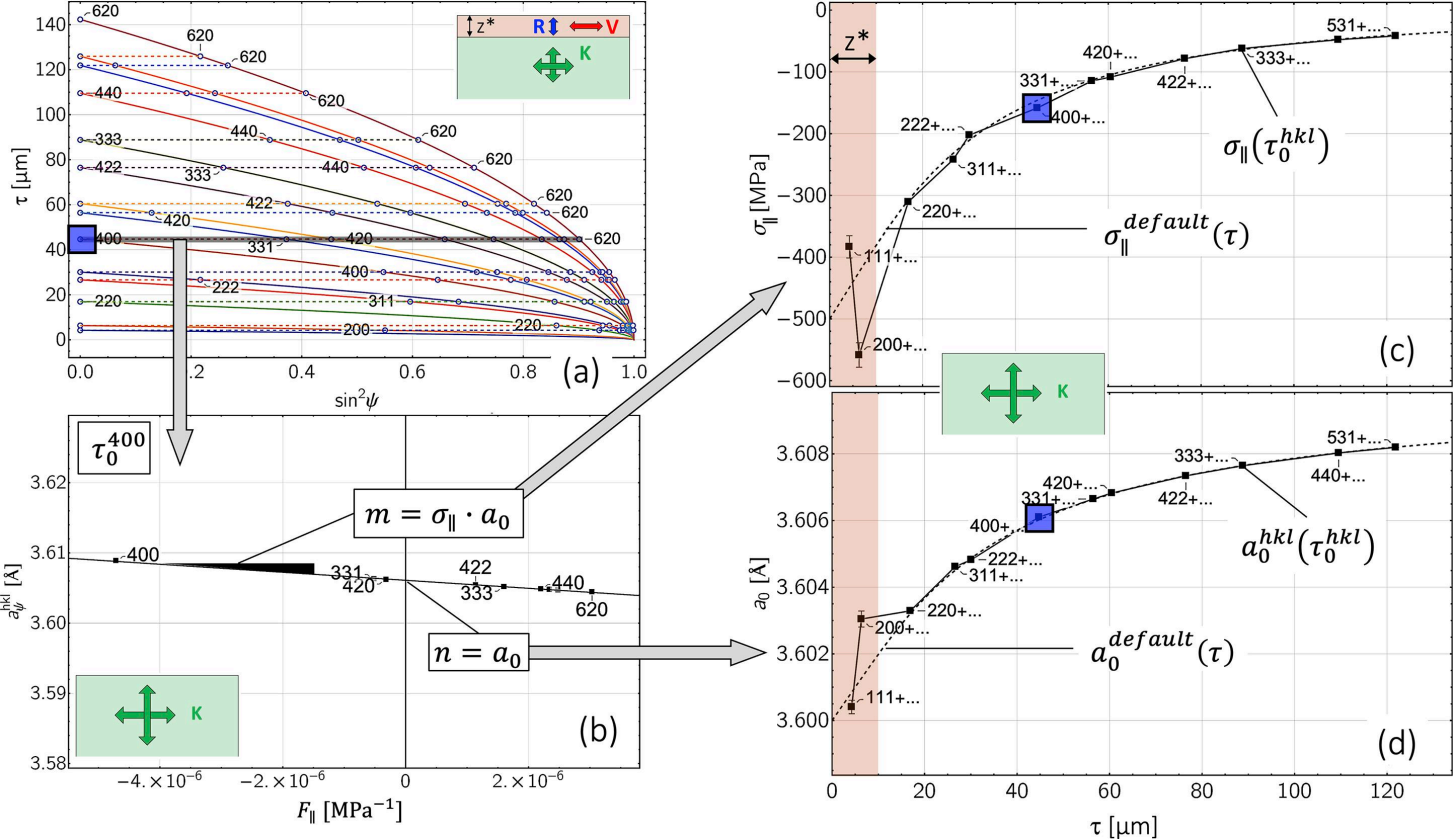
Data analysis by means of the constant-τ method. The underlying sin^2^ψ data are the same as in Fig. 6 ▸(*a*). For reasons of clarity, only selected points *hkl* are indexed in diagram (*a*). The notation *hkl* + … in diagrams (*c*) and (*d*) is intended to indicate that, in addition to the reflection *hkl*, all other reflections with higher photon energy >*E*^*hkl*^ also contribute to the evaluation of the associated data point. See text for further details.

The constant-τ method is based on the stress factor concept that was developed (Dölle & Hauk, 1978 ▸, 1979*b* ▸) and applied [see *e.g.* Baczmanski *et al.* (2003 ▸)] for XSA in highly textured materials. It allows the separation of residual stress and composition depth gradients in thin films (Klaus & Genzel, 2017 ▸; Klaus *et al.*, 2017 ▸) and bulk materials (Marciszko-Wiackowska *et al.*, 2019 ▸). The principal procedure for data evaluation is shown in Fig. 7 ▸. We consider the same data as in Fig. 6 ▸(*a*). The generation of data sets at constant depths 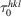 shall be explained using the example of the 400 reflection [*cf*. Fig. 7 ▸(*a*)]. In order for all higher-energy reflections *hkl* (*i.e.E*^*hkl*^ > *E*^400^) to originate from the same depth 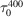, the following condition must be met: 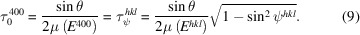
This provides the ψ^*hkl*^ values for which the (normalized) *d* spacing *a*^*hkl*^ originates from the predefined information depth 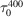: 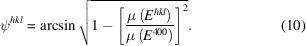
In this way, a data set 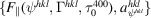 can be generated, from which the in-plane residual stress σ_∥_ and the strain-free lattice parameter *a*_0_ for the depth 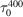 can be determined using linear regression according to equation (7) [*cf*. Fig. 7 ▸(*b*)]. In this evaluation, instead of the actual (depth-dependent) stress factors 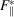 [*cf*. equation (5)], we use the volume stress factors 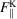 according to equation (4*b*[Disp-formula fd4b]) as arguments for the fit function in order to quantify the influence of the grain interaction model, and to calculate the differences from the default stress and composition depth profiles, 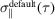 and 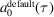, respectively. For our example with the 400 reflection, the fit function therefore reads
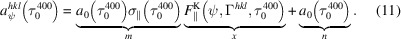
The value 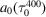 can thus be determined directly from the ordinate intercept *n* of the regression line and the value for the stress 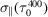 can then also be determined from the slope *m*. If this procedure is continued for all other evaluable section depths 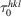, the discrete depth profiles shown in diagrams (*c*) and (*d*) of Fig. 7 ▸ are obtained.

## 4. Case studies

### 4.1. Objective and approach

**Figure 8:**
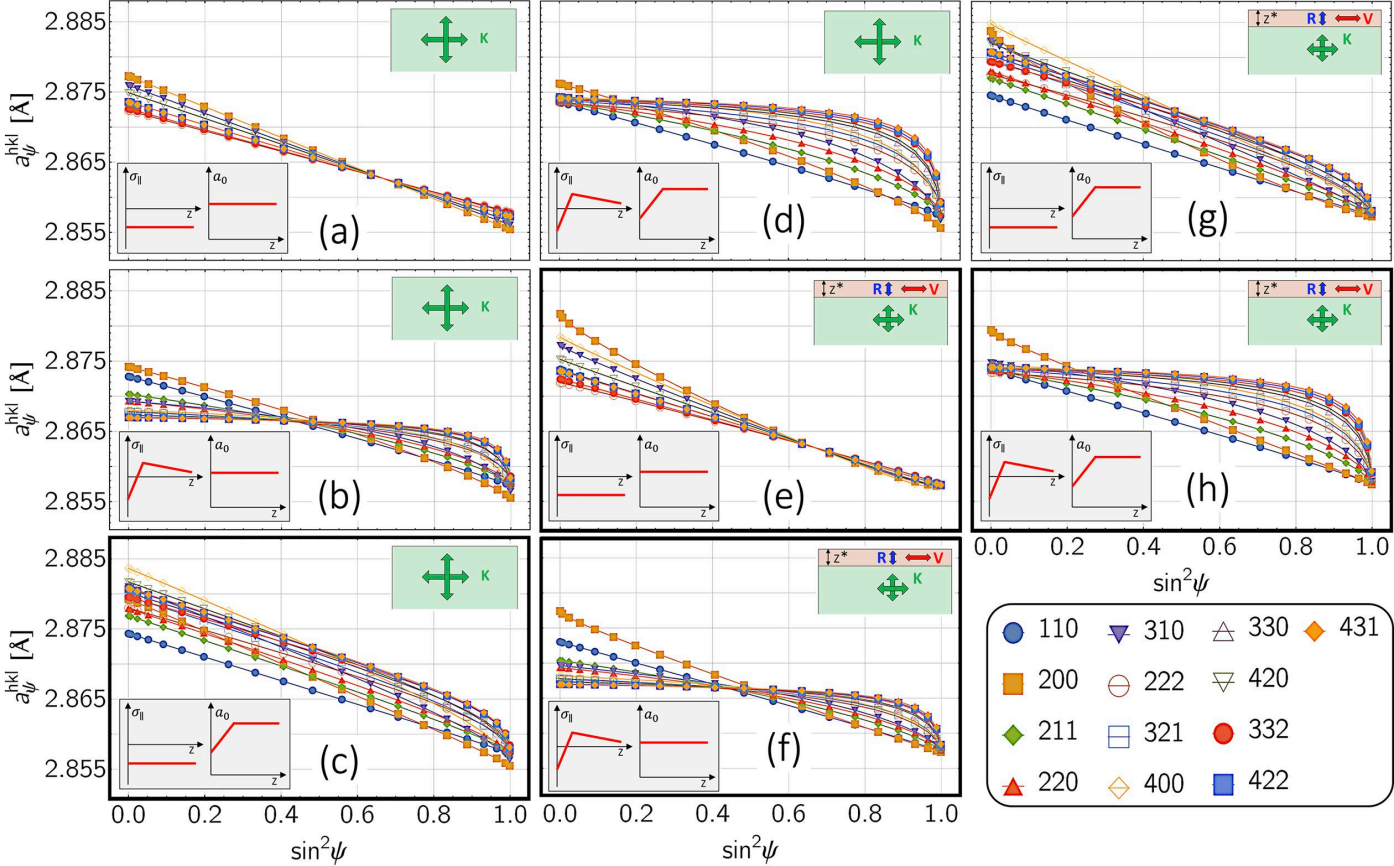
Normalized 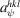
–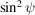 plots for different scenarios concerning the superposition of uniform and non-uniform depth distributions of stress, composition and grain interaction in the near-surface region of a ferritic steel. The calculations are based on the depth profiles for the stress and the strain-free lattice parameter shown in Fig. 9 ▸, and were carried out for a diffraction angle 2θ = 16° (*cf*. Table 1 ▸) and 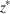 = 20 µm for the cases (*e*)–(*h*).

**Figure 9:**
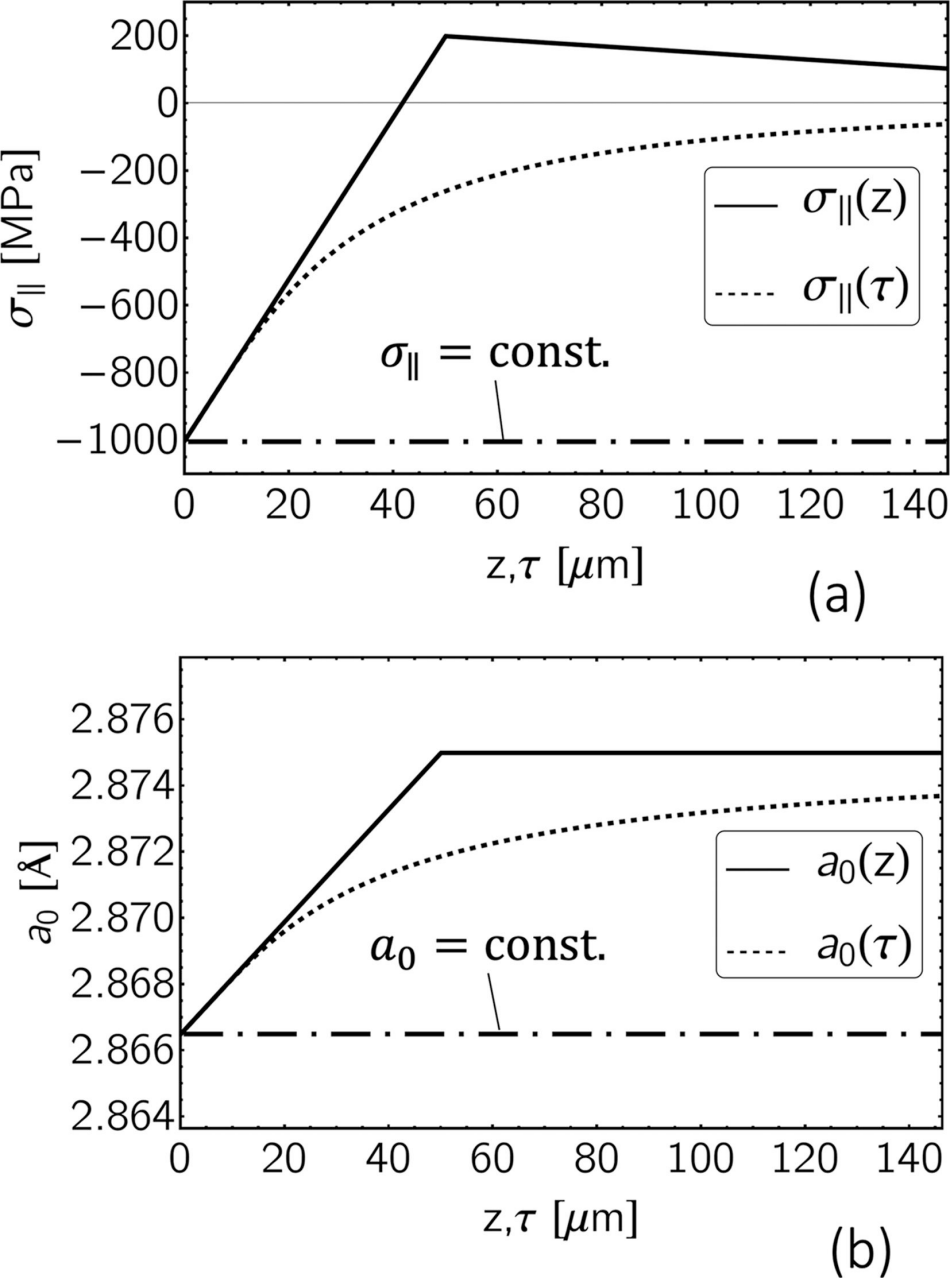
Residual stress (*a*) and lattice parameter depth profiles (*b*), on which the sin^2^ψ distributions in Fig. 8 ▸ are based. For the depth profiles assumed to be homogeneous with depth (dash–dotted lines), the real- and Laplace-space distributions coincide.

In the following, the influence of near-surface grain interaction and material composition on the X-ray residual stress analysis will be investigated. The aim of this study is in particular to show how the influence of the generally unknown depth-dependent grain interaction, which is difficult to determine experimentally, on the results of the analyses can be bypassed. For this purpose, the elastically anisotropic surface layer 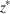 (see Fig. 2 ▸) assumed in the generation of the sin^2^ψ distributions is neglected in the evaluation using the methods considered here. The broad matrix of parameters that could be varied for this purpose (thickness 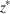, diffraction angle 2θ, information depth τ, steepness and presence or absence of the stress and composition depth gradients *etc*.) will be limited to a few particularly important cases which are marked in Fig. 8 ▸ by thick borders. The 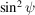 distributions are based on the residual stress and lattice parameter depth profiles shown in Fig. 9 ▸. We emphasize that the specific form of the gradients for stress and composition has no impact on the key messages of the paper, which aim to show the impact of gradients in general on the results of the two XSA methods. Other combinations of depth profiles, for example, an increase in compressive stresses at depth with a simultaneous decrease in the strain-free lattice parameter, which is also an important case from a practical point of view, will therefore not be considered here.

From cases (*a*) to (*d*), in which a homogeneous grain interaction model according to Eshelby–Kröner is assumed, only case (*c*) is investigated in more detail, as it allows important conclusions to be drawn about the applicability of the two data evaluation methods considered in the present paper. The ‘trivial’ cases (*a*) (analysis by the conventional sin^2^ψ method) and (*b*) [evaluation possible using the stress gradient methods that have been described in the literature; see *e.g.* Genzel *et al.* (2013 ▸)], on the other hand, are not considered in detail. The same applies to cases (*d*) and (*g*), which can be discussed in connection with the related cases (*h*) and (*c*), respectively.

All simulations were carried out for a diffraction angle 2θ = 16°, since for this angle the highest-intensity reflections *hkl* for both ferrite and austenite are in an energy range that can also be realized with laboratory X-ray sources (see Table 1 ▸). The relationship between the position of the diffraction lines *E*^*hkl*^ on the energy scale, the diffraction angle 2θ and the normalized lattice spacings *a*^*hkl*^ = 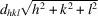 (for cubic crystal symmetry) is given by Bragg’s equation for ED diffraction (Giessen & Gordon, 1968 ▸; Buras *et al.*, 1968 ▸):
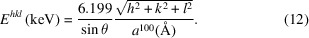
Double-indexed reflections (*e.g.* 330/411 for ferrite or 333/511 for austenite) (for reasons of clarity, only the first index is given in all graphs in this paper) cannot be included in the evaluation without further consideration, as the respective lattice directions differ in their elastic behavior (*i.e.* their orientation 3Γ^*hkl*^, see Table 1 ▸). Appendix *C*[App appc] shows that these diffraction lines split on the energy scale in addition to the stress-induced absolute shift and thus get an asymmetric shape.

### 4.2. Ferritic steel, stress gradient without and with superimposed composition gradient

**Figure 10:**
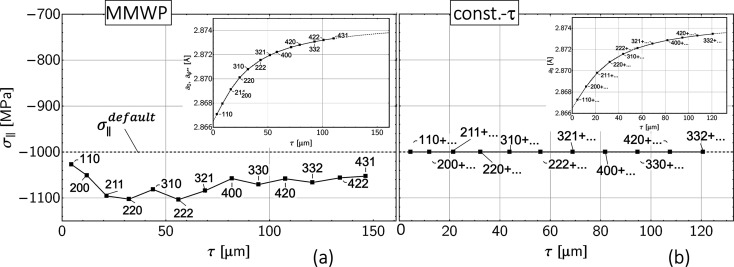
Analysis of the sin^2^ψ data shown in Fig. 8 ▸(*c*) by (*a*) the modified multi-wavelength plot method and (*b*) the constant-τ method. The default profiles in Laplace space are indicated by dashed lines in the diagrams above and all the following diagrams. The insets show the depth profiles obtained for the strain-free lattice parameter.

The first example refers to the case shown in Fig. 8 ▸(*c*). Since an influence of both stress and grain interaction gradient on the 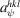
–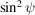 distributions has been excluded because σ_∥_ = constant and 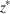 = 0, the curvatures in diagram (*c*) at large ψ angles and increasing photon energies *E*^*hkl*^ are solely due to the influence of the composition (*i.e.a*_0_) gradient. The results of the data analysis by the methods described in Sections 3.1 and 3.2 are summarized in Fig. 10 ▸.

For this scenario, the MMWP method provides a discrete depth profile for the in-plane residual stress that is systematically shifted compared with the specifications [Fig. 10 ▸(*a*)]. This finding can be explained by the fact that the MMWP method is an integrating method that derives its information content from exponentially weighted averaging. Therefore, it is not possible to distinguish whether the slope and the nonlinearities of the 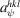
–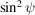 distributions originate from a stress or the *a*_0_ gradient. Note that the deviation of the stresses from the default scales directly with the absolute stress level. This means that the smaller the stresses are in the accessible depth range, the larger the relative error becomes. The stress difference 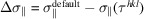 may be regarded as ‘ghost stresses’ which have been extensively reported in the literature. An AD diffraction method based on stepwise layer removal that allows one to separate residual stress and composi­tion depth gradients and to quantify the ghost stresses was introduced by Somers & Mittemeijer (1990 ▸) and later com­pared with the classical sin^2^ψ-based evaluation approach by Christiansen & Somers (2006 ▸). In the latter paper, as in the present work, the authors used simulations to demonstrate that a sin^2^ψ-based evaluation may only be applied if there are no *a*_0_(*z*) gradients within the depth range covered by the X-ray beam.

In contrast, the results of the constant-τ method in Fig. 10 ▸(*b*) match the specifications very well. This result can also be attributed to the nature of the method, which consists of analyzing data sets from well defined, constant depths below the surface, where the gradient nature of the stress and composition profiles does not have any influence. The suitability of constant-τ-based measuring and data evaluation techniques for a nondestructive separation of stress and composition depth gradients has also been confirmed by other authors. Using an AD grazing-incidence diffraction technique (Fernandes *et al.*, 2017 ▸) and high-energy ED diffraction in combination with the scattering vector method (Jegou *et al.*, 2013 ▸), respectively, *a*_∥_(*z*) and *a*_0_(*z*) gradients could be separated successfully in the near-surface region of expanded austenite.

Finally, the depth profile for the strain-free lattice parameter *a*_0_ is correctly reproduced for both methods. For the constant-τ method, this result is obvious since the influence of the *a*_0_ gradient is eliminated by evaluating predefined depths. In the case of the MMWP method, this finding can be explained by the fact that the sin^2^ψ distributions remain linear even in the presence of steep gradients up to about sin^2^ψ = 0.5 (Klaus & Genzel, 2019 ▸). For a biaxial stress state, *a*_0_ can therefore be determined to a good approximation from the strain-free direction 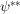. Moderate deviations from the default *a*_0_ profile only occur if there is a grain interaction gradient in the near-surface zone, but this is not taken into account in the analysis (see Fig. 6 ▸). In the example shown there the maximum deviation observed for the reflection 200 is 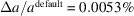.

**Figure 11:**
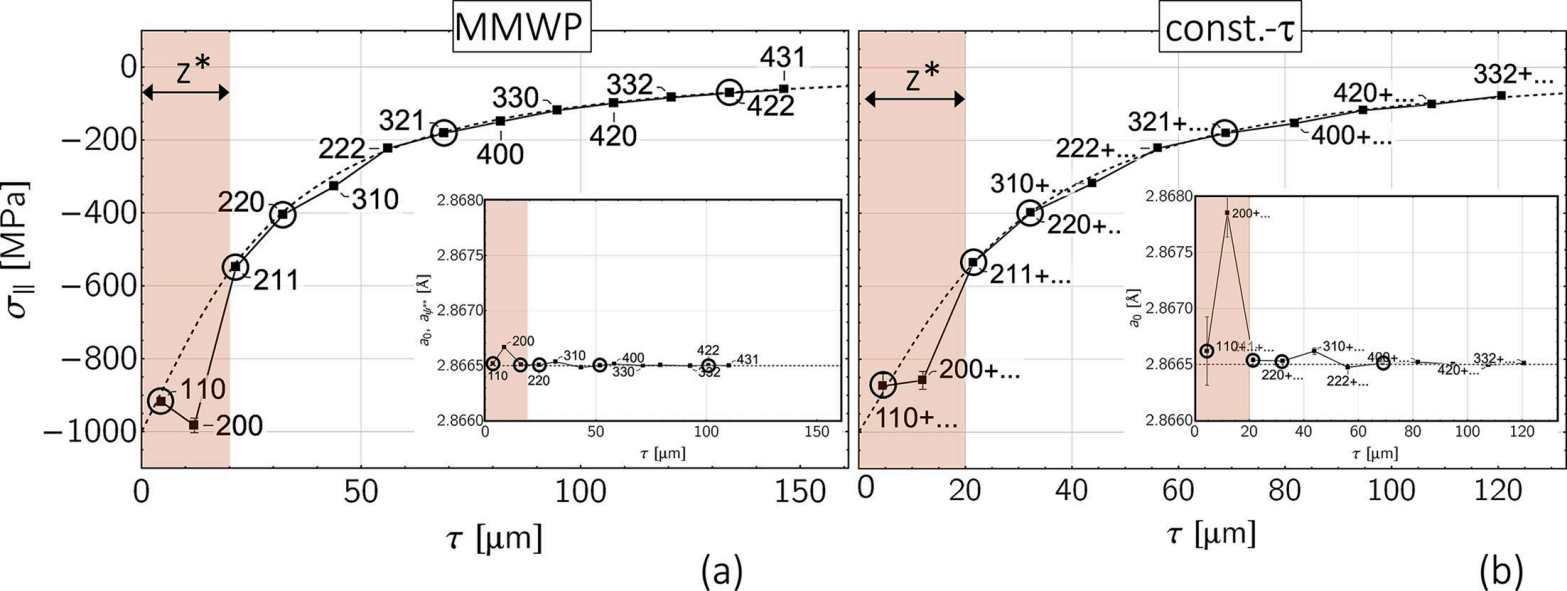
Analysis of the sin^2^ψ data shown in Fig. 8 ▸(*f*) by (*a*) the modified multi-wavelength plot method and (*b*) the constant-τ method. The thickness of the anisotropic surface layer (gray area) was assumed to be 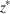 = 20 µm. The circled data points denote reflections whose orientations 3Γ^*hkl*^ are close to the model-independent orientation 3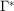 (see Table 1 ▸).

The reverse case in the form of a pronounced stress depth gradient but a constant strain-free lattice parameter is considered in Fig. 11 ▸ [*cf*. diagram (*f*) in Fig. 8 ▸]. In addition, a 20 µm-thick surface layer 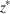 with anisotropic grain interaction was assumed. The results reveal for both methods a very good agreement of the evaluated discrete stress and composition depth profiles with the default profiles. The only exception is the 200 reflection, whose orientation 3Γ^200^ = 0 is furthest away from 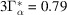 (*cf*. Fig. 18). In addition, because 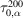 = 12 µm, its information content originates largely from the anisotropic surface layer 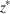, which was neglected in the evaluation.

**Figure 12:**
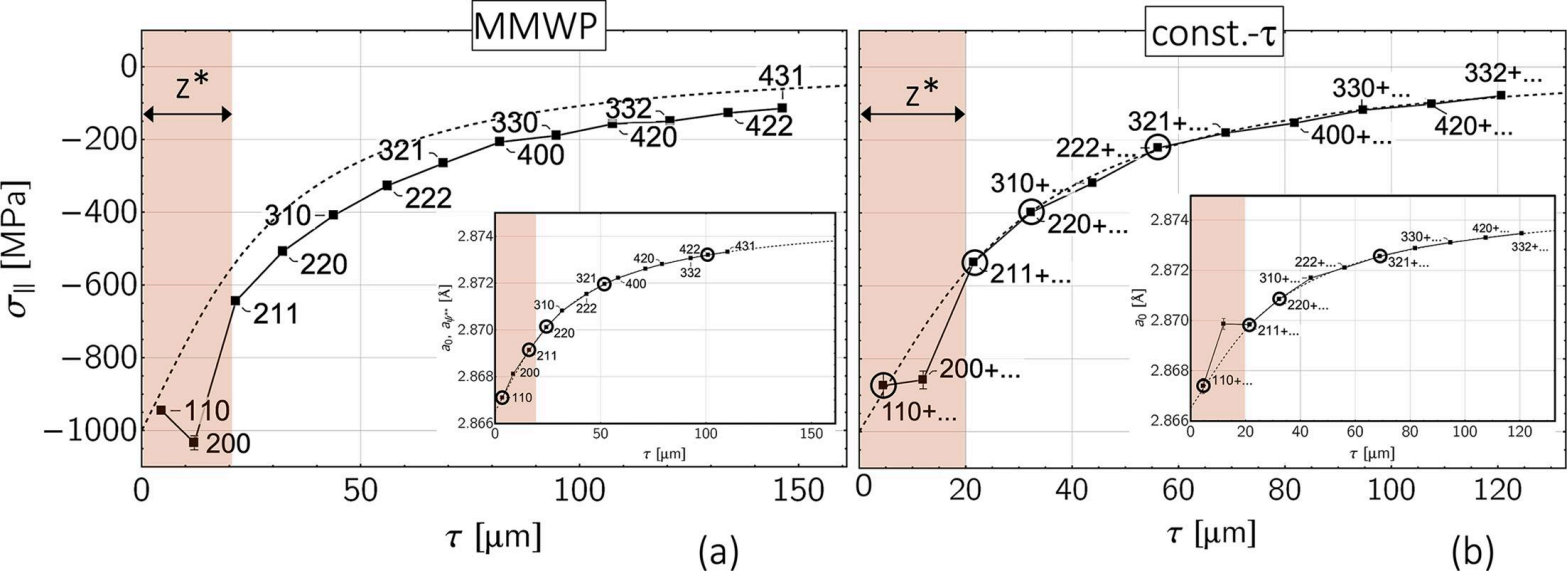
Analysis of the sin^2^ψ data shown in Fig. 8 ▸(*h*) by (*a*) the modified multi-wavelength plot method and (*b*) the constant-τ method. See Fig. 11 ▸ for an explanation of the symbols.

The results for the superposition of all three depth gradients [stress, composition, grain interaction; *cf*. diagram (*h*) in Fig. 8 ▸] are shown in Fig. 12 ▸. The comparison of the two evaluation methods confirms the finding from Fig. 10 ▸ that a correct separation of stress and composition gradients is only possible using the constant-τ method. In addition, the systematic shift of the stress values determined with the MMWP method parallel to the abscissa axis indicates that they may only be plotted versus 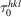 if the strain-free lattice parameter *a*_0_ is not depth dependent (Klaus & Genzel, 2019 ▸), which is not the case in the present example.

### 4.3. Comparison of ferritic and austenitic steel, impact of grain interaction

**Figure 13:**
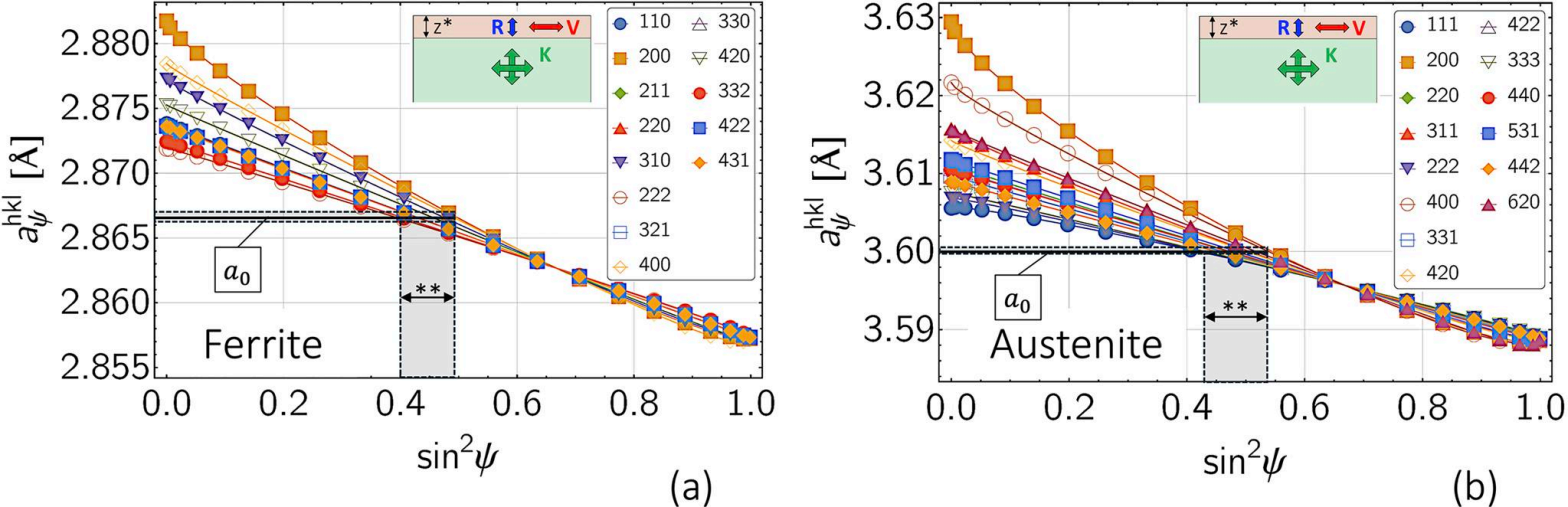
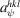
–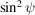 distributions for (*a*) ferritic and (*b*) austenitic steel which correspond to an in-plane residual stress σ_∥_ = −1000 MPa and uniform strain-free lattice parameters *a*_0_. The gray areas mark the range of angles 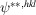 under which the strain-free lattice parameters 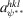 are obtained for the biaxial stress state. The thickness of the anisotropic surface layer was assumed to be 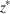 = 20 µm.

In this section, we investigate the impact of elastic single-crystal anisotropy and depth-dependent grain interaction on the results of the stress analysis. To illustrate the influence of these two factors, 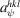
–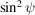 distributions are considered for a ferritic and an austenitic steel, which result for a residual stress and composition state which is homogeneous within the information depth of the X-rays. Under these conditions, the curvature of the sin^2^ψ distributions is solely caused by the anisotropic surface layer (see Fig. 13 ▸). Note that the high (constant) stress level of −1000 MPa was only selected for comparison with the other examples considered in the previous section. It may be realistic for ferrite/martensite but should be too high for austenite in most cases.

**Figure 14:**
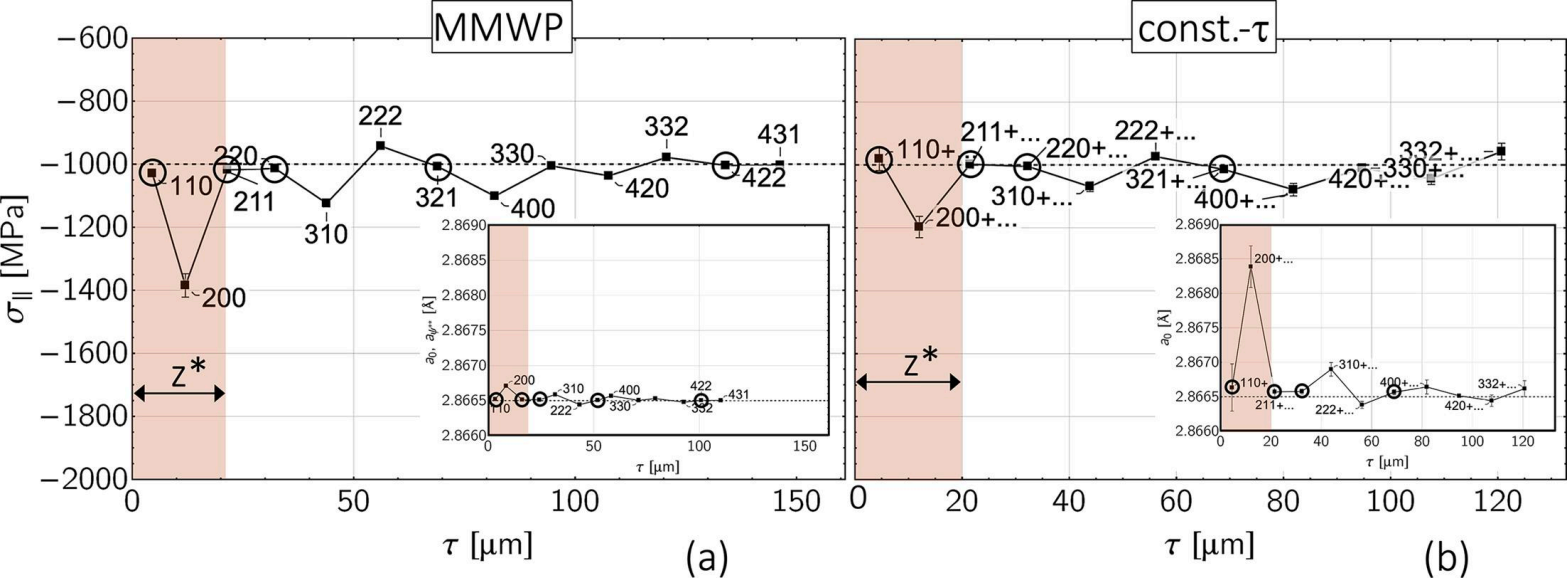
Analysis of the sin^2^ψ data for the ferritic steel shown in Fig. 13 ▸(*a*) by (*a*) the MMWP method and (*b*) the constant-τ method. The evaluation was carried out under the assumption that the Eshelby–Kröner grain interaction model is valid within the entire depth range covered by the X-ray beam.

The results of the data analysis obtained by means of the MMWP method and the constant-τ method are compared in Figs. 14 ▸ (ferrite) and 15 ▸ (austenite). Since depth gradients were excluded for both the residual stresses and the composition, the deviations of both depth profiles, 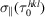 and 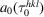, from the default values can be attributed solely to the influence of the depth-dependent grain interaction. The evaluation for both materials reveals that the maximum deviations occur in the region of the anisotropic surface layer 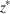 and there especially for the 200 reflection. The reason for this is that, on the one hand, the information depths 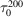 are smaller than 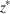 for both materials (see Table 1 ▸) and that, on the other hand, the DECs in this crystal direction feature the strongest differences between the grain interaction models according to Voigt, Eshelby–Kröner and Reuss because 3Γ^200^ = 0 (*cf*. Fig. 18).

On the absolute scale, due to the larger single-crystal anisotropy ratio of austenite (*A*_γ_ = 3.5) compared with ferrite (*A*_α_ = 2.4), significantly larger deviations of the evaluated stress depth profiles, 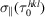, from the default value are observed for austenite. What both materials and XSA evaluation methods have in common is that reflections with a higher information depth provide results that are closer to the default, as the influence of the anisotropic surface layer becomes increasingly smaller with increasing depth. It is in addition characteristic that reflections with *hkl* close to the model-independent orientation 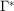 invariably yield stress values close to the specification. This also applies to the diffraction lines with small information depths, 110_α_ and 211_α_ for ferrite, and 220_γ_ in the case of austenite.

Regardless of the material, the simulations also show characteristic differences between the two approaches used for the evaluation. The 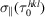 depth distributions reveal that the constant-τ method provides results that are closer to the default on average. The reason for this finding is that the constant-τ method derives its information content from the evaluation of lattice strains which originate from a constant depth below the surface. For each predefined depth, various reflections *hkl* contribute to the regression line, some of which are also close to the model-independent orientation 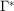 [see Fig. 7 ▸(*b*)]. As a result, the influence of the ‘incorrect’ volume grain interaction model used in the evaluation is partially or even completely averaged out. In contrast, the MMWP method averages over a more or less large depth range for only one single diffraction line. If these reflections *hkl* are linked to crystal lattice directions with an unfavorable orientation Γ^*hkl*^ far from the model-independent orientation, the stress values calculated from the corresponding sin^2^ψ regression line will feature a larger deviation from the default value.

**Figure 15:**
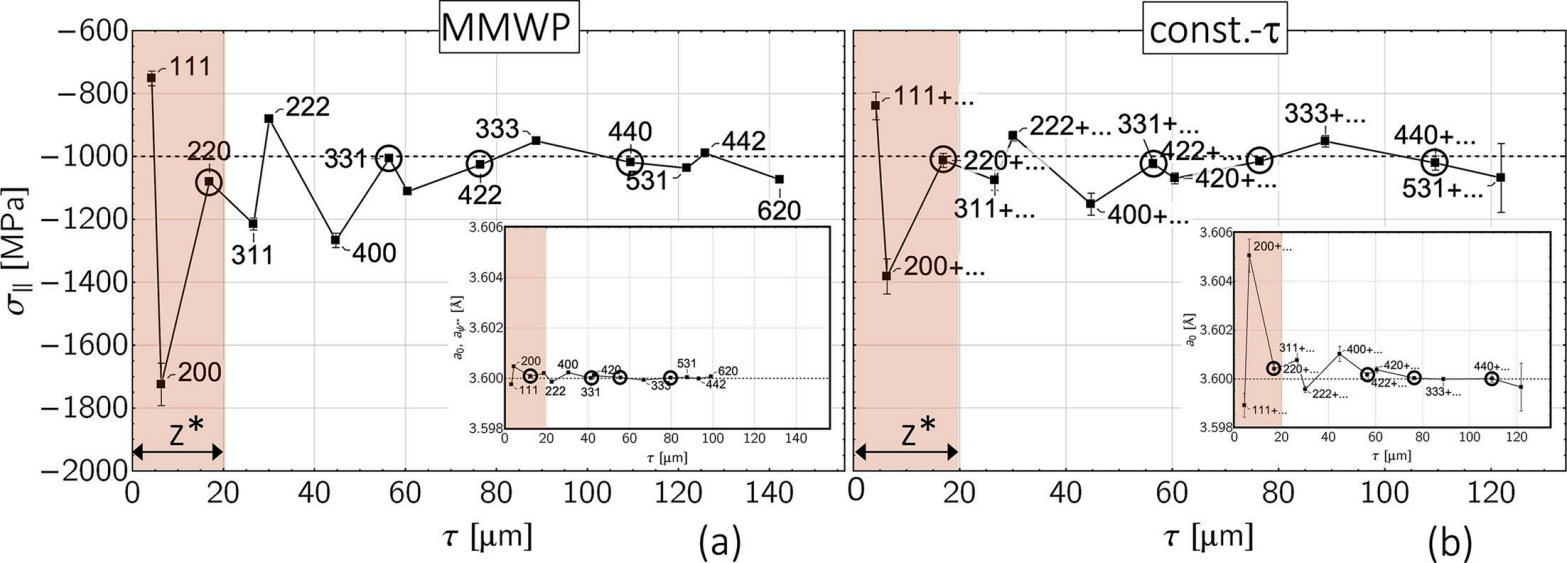
Analysis of the sin^2^ψ data for the austenitic steel shown in Fig. 13 ▸(*b*) by (*a*) the MMWP method and (*b*) the constant-τ method, based on the Eshelby–Kröner grain interaction model (*cf*. Fig. 14 ▸).

Concerning the depth profiles *a*_0_(τ), the maximum differences between the defaults and the values obtained in the data evaluation are rather small for both methods considered here. Even under the worst-case conditions [constant-τ method, 200+ reflection, see inset in Fig. 15 ▸(*b*) and Fig. 17] the shift Δ*a*/*a*^default^ does not exceed 0.14%.

### 4.4. Refinement of the depth-dependent grain interaction model

**Figure 16:**
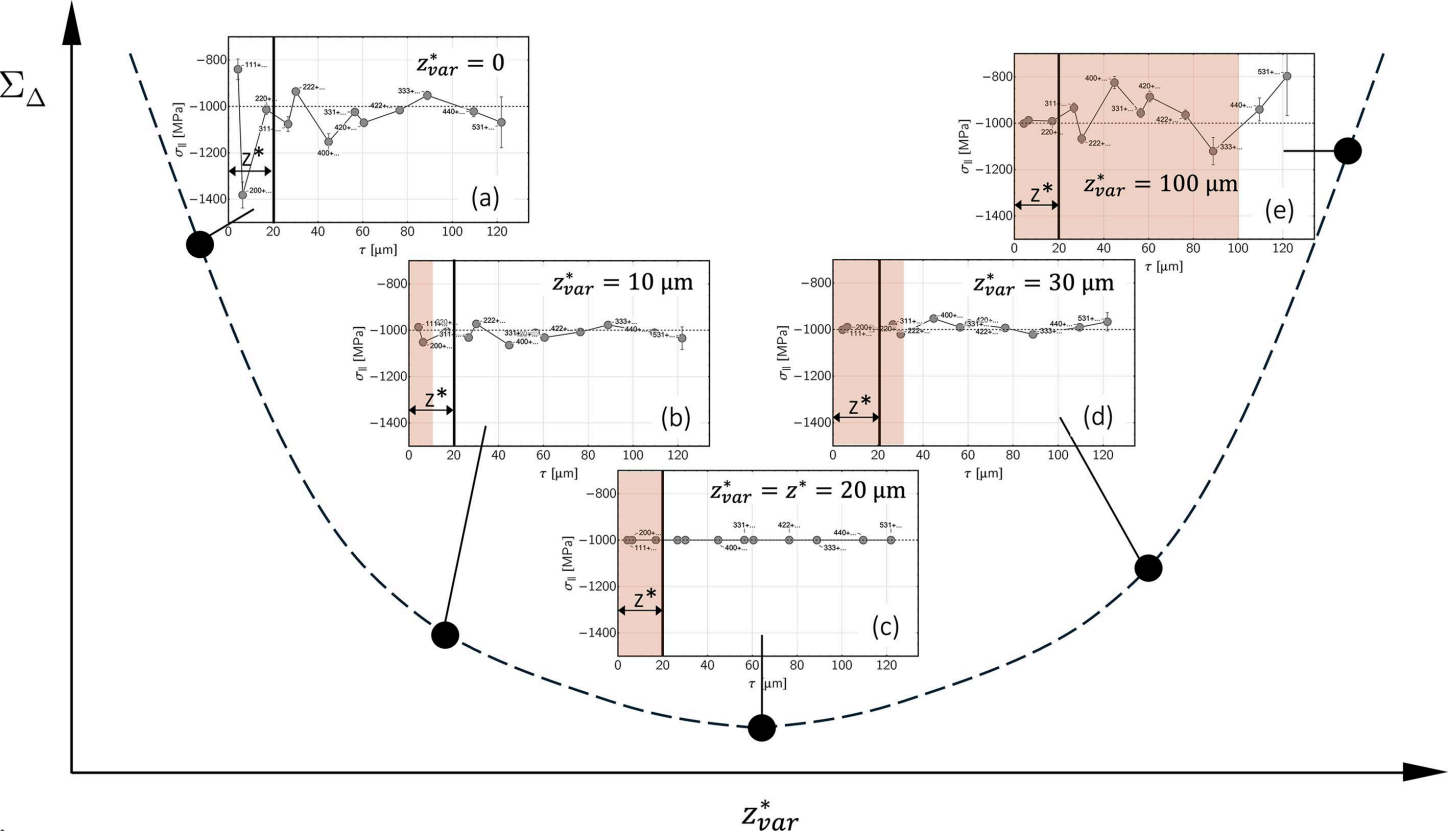
The optimization procedure for the refinement of the grain interaction model. See text for details.

We apply the optimization method introduced by Klaus & Genzel (2019 ▸) to determine a homogeneous DEC model to the case of depth-dependent grain interaction. The procedure is illustrated in Fig. 16 ▸. The example refers to austenitic steel which should feature a homogeneous in-plane residual stress and depth-dependent grain interaction such as that introduced in Fig. 2 ▸. The thickness of the anisotropic surface layer is assumed to be 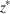 = 20 µm. The data analysis is based on the constant-τ method, because, as shown in the previous section, it provides correct results for the stress depth profiles, 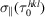, also if composition depth gradients are present in the material’s near-surface region (*cf*. Fig. 7 ▸).

In contrast to the analysis outlined in the previous sections, data evaluation is now performed by taking into account the depth dependency of the grain interaction model. The parameter that is varied during the optimization procedure is the thickness of the anisotropic surface layer. Thus, if one performs data analysis for a set of values 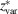 taken for 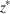 in equation (5), the obtained 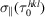 profiles will feature more or less strong jumps between neighboring points when 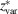 is far away from the actual 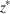 [diagrams (*a*) and (*e*) in Fig. 16 ▸]. The closer 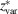 approaches 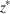, the smoother the stress depth profiles will become [diagrams (*b*) and (*d*)], until the point 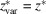 is reached [diagram (*c*)]. This point corresponds to the minimum path length 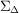 defined by equation (13), which describes the sum of straight lines connecting neighboring points of the 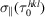 profile, 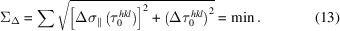
The procedure outlined above is based on the assumption that stresses σ(*r*) in the Laplace space should feature no jumps but rather smooth profiles as they are the exponentially weighted response to the actual σ(*z*) profiles in the real space. The search for the minimum of 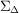 therefore means finding the value of 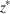 that best describes the depth-dependent grain interaction in the material region close to the surface. Genzel *et al.* (2023 ▸) applied this formalism to experimental data obtained for an austenitic steel in order to refine the weighting factor *r* which describes a homogeneous grain interaction model between the Voigt and Reuss limits according to equations (16*a*[Disp-formula fd16a]) and (16*b*[Disp-formula fd16b]). Therefore, the study of Genzel *et al.* (2023 ▸) (see Fig. 1 ▸) and the case shown in Fig. 16 ▸ differ because the latter assumes that the grain interaction models in the surface layer and the volume are known and the parameter to be refined is the thickness of the anisotropic surface layer.

## 5. Discussion

In the present work, simulated data were used in order to evaluate the suitability of two different XSA methods for separating the influence of various combinations of stress, composition and grain interaction gradients in the near-surface material region. The advantage of simulations is that the correct solution is known, which is not the case with real experiments. This approach seems justified since the methods investigated here for analyzing ED diffraction data sets are already being used successfully in practice. This work therefore addresses other issues. The focus is on the important question from the user’s perspective of the extent to which the non-consideration of influencing factors that are difficult to check, such as a depth-dependent grain interaction, leads to errors in stress analysis and to show ways in which these uncertainty factors can be bypassed. Table 2 ▸ summarizes the findings in this regard.

The methods compared in the table both access the same sin^2^ψ measurements. However, they differ fundamentally in their evaluation strategy. According to equation (6) the MMWP method derives its information content from the evaluation of data originating from different depths 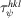 of the total depth range covered by the X-ray beam. Therefore, it might be considered an ‘integral method’ that fails if a (significant) depth gradient of the strain-free lattice parameter *a*_0_ is superimposed on the stress (gradient) [cases *a*_0_ = *f*(*z*) in Table 2 ▸] since its contribution to the slope of the sin^2^ψ distributions is (mis)interpreted as stress. The constant-τ method might be considered a ‘local method’ since the data used for the analysis of the stress and the strain-free lattice parameter originate from predefined depths below the surface. Thus, the two superimposed gradients can be separated.

**Figure 17:**
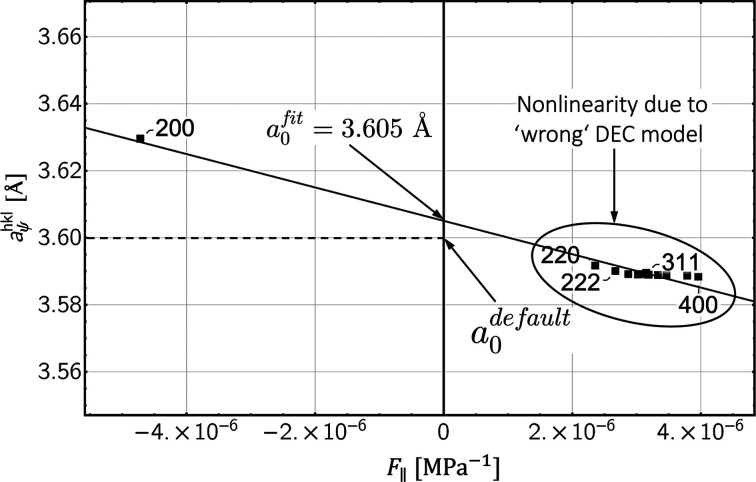
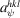
–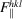 plot, the evaluation of which provides the data points 200 + … in Fig. 15 ▸(*b*).

**Figure 18:**
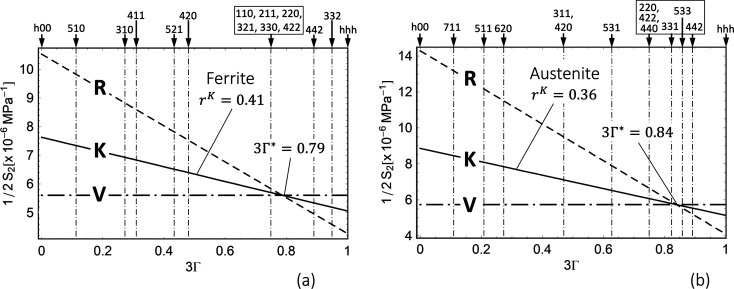
Diffraction elastic constant 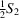 for (*a*) ferritic and (*b*) austenitic steel, calculated by means of equations (14*a*[Disp-formula fd14a]), (14*b*[Disp-formula fd14b])–(16*a*[Disp-formula fd16a]) and (16*b*[Disp-formula fd16b]) for the grain interaction models of Reuss (R), Voigt (V) and Eshelby–Kröner (K). The framed reflections *hkl* lie in the vicinity of the model-independent orientation 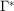. *r*^K^ denotes the Reuss fraction in the Eshelby–Kröner DEC model. The single-crystal elastic moduli *s*_*ij*_ were taken from Landoldt–Börnstein (1984 ▸).

In general it holds true that the larger the information depth, the better both the σ_∥_(τ) and the *a*_0_(τ) depth profiles are reproduced by both methods, because the high-energy reflections are less affected by the anisotropic surface layer 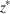. However, it is striking that the *a*_0_ values determined using the constant-τ method feature larger deviations from the standard profile for <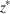. There are two reasons for this finding, which are explained in Fig. 17 ▸. (i) The information for small τ originates completely from depths where the ‘wrong’ grain interaction model is used for the analysis. This leads to the nonlinearity in the 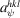
–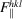 plot marked in the diagram. (ii) For small information depths there is only one negative stress factor compared with many positive factors on the other side in the 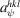
–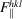 plots, which leads to an unfavorable ‘leverage effect’ in the least-squares fit.

The issue of depth-dependent grain interaction [cases *F*_∥_ = *f*(*z*) in Table 2 ▸] can be bypassed with the constant-τ method and the MMWP method [the latter only for the case *a*_0_ ≠ *f*(*z*)], if the analysis is confined to reflections *hkl* with orientation factors Γ^*hkl*^ close to the model-independent orientation 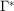. However, this is at the expense of the number of nodes in the stress depth profile, which is more problematic for austenitic steel compared with ferrite, as the two near-surface reflections 111_γ_ and 200_γ_ do not fulfill the condition Γ^*hkl*^ ≃ 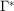 [see Fig. 15 ▸ but also Fig. 1 ▸(*c*)]. One way of estimating the thickness 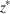 of the anisotropic surface layer was outlined in Fig. 16 ▸.

Note that the optimization procedure in Fig. 16 ▸ was not used to refine grain interaction models themselves. With the Eshelby–Kröner model for the volume, however, an assumption was made that appears reasonable for materials with a random texture and has often been confirmed experimentally by load–stress measurements. For the anisotropic surface layer, extreme assumptions according to Reuss (Voigt) were made with the free (fully constrained) deformation perpendicular (parallel) to the surface. These critical assumptions aimed to demonstrate the maximum impact of a depth-dependent grain interaction on the ED X-ray stress analysis.

Some further points that are important from the user’s point of view should be noted. All considerations in this paper were made for a biaxial, rotationally symmetrical stress state. However, this is not a limitation, as in the case of a non-rotationally symmetric stress state only measurements under two azimuths φ = 0° and φ = 90° need to be combined [see *e.g.* Genzel *et al.* (2013 ▸)]. The answer to the question of which of the two XSA methods discussed here is the more appropriate depends on the material condition in the near-surface region. If gradients of the strain-free lattice parameter can be excluded, the MMWP method should be applied, as sin^2^ψ measurements up to about ψ = 45° are usually sufficient for data analysis. The constant-τ method, on the other hand, requires measurements up to very large ψ angles and should therefore only be used if a superposition of stress and (pronounced) composition gradients is to be expected.

Finally, the analysis in this paper was restricted to the determination of the discrete stress depth profiles in the Laplace space, 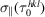. For the inverse transform into the real space, which provides the actual depth profiles σ_∥_(*z*), the reader is referred to the literature. Methods often used in practice in this respect are based on the description of the real-space profiles by polynomial functions with (Hauk & Krug, 1988 ▸) and without (Ruppersberg *et al.*, 1991 ▸; Denks *et al.*, 2009 ▸) exponential damping, whose Laplace transforms are adapted to the experimental depth profiles employing a least-squares fit. An approach based on the inverse numerical Laplace transform that allows the direct calculation of the σ_∥_(*z*) profiles from the discrete 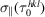 distributions was suggested by Genzel (1996 ▸).

## Figures and Tables

**Figure 19:**
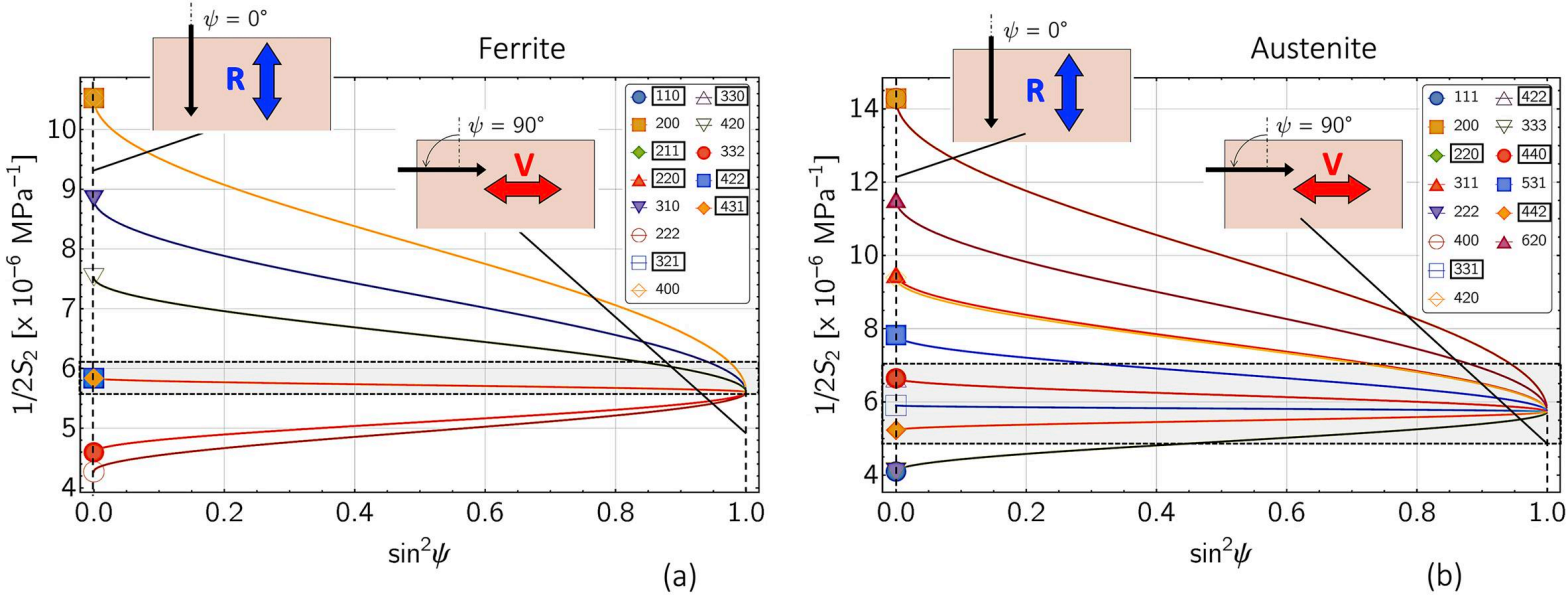
Direction dependency of the diffraction elastic constant 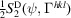 according to equation (18*b*[Disp-formula fd18b]) for (*a*) ferritic and (*b*) austenitic steel. The framed *hkl* denote reflections featuring a Γ^*hkl*^ close to the model-independent orientation 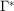 (*cf*. Fig. 18 ▸).

**Figure 20:**
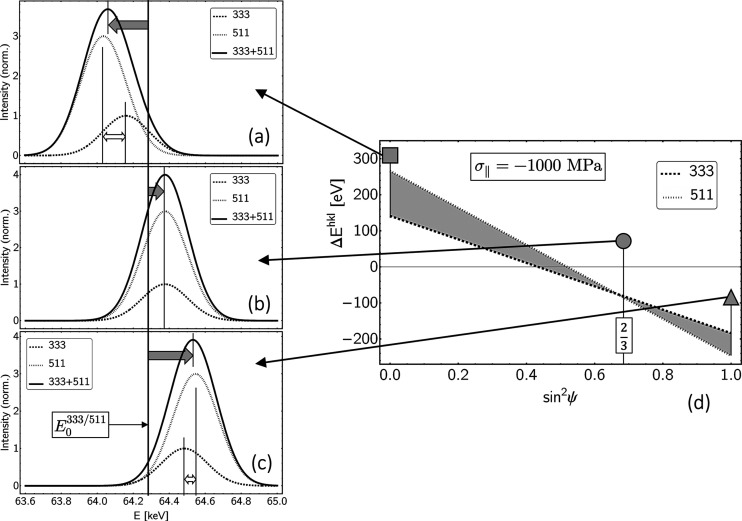
(*a*)–(*c*) Absolute shift (filled arrows) and splitting (empty arrows) of the 333/511 diffraction line for an austenitic steel. The line position 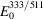 = 64.3 keV for the strain-free lattice corresponds to a diffraction angle 2θ = 16° (*cf*. Table 1 ▸). Diagram (*d*) shows the absolute shift of the 333 and 511 contributions as a function of sin^2^ψ according to equation (19) and their relative shift against each other (gray area between the straight lines) according to equation (20). The calculation was done assuming the Eshelby–Kröner grain interaction model.

**Table 1:** Diffraction line position *E*^*hkl*^, maximum information depth sin^2^ψ and the triple orientation factor 3Γ^*hkl*^ for ferritic (α) and austenitic (γ) steel for 2θ = 16° The bold values of 3Γ^*hkl*^ refer to reflections close to the model-independent orientations 3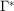, which are 0.79 and 0.84 for ferritic and austenitic steel, respectively (*cf*. Fig. 18 ▸).

*hkl*_α_	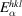 (MPa)	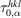 (µm)	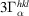	*hkl*_γ_	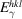 (MPa)	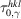 (µm)	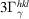
110	22.0	4.5	**0.75**	111	21.4	4.2	1.00
200	31.1	12.0	0.00	200	24.7	6.3	0.00
211	38.1	21.5	**0.75**	220	35.0	16.9	**0.75**
220	43.9	32.3	**0.75**	311	41.0	26.3	0.47
310	49.1	43.9	0.27	222	42.9	30.1	1.00
222	53.8	56.1	1.00	400	49.5	44.7	0.00
321	58.1	68.9	**0.75**	331	53.9	56.5	**0.82**
400	62.1	81.9	0.00	420	55.3	60.5	0.48
330/411	65.9	94.7	0.75/0.31	422	60.6	76.5	**0.75**
420	69.5	107.6	0.48	333/511	64.3	88.8	1.00/0.21
332	72.9	120.7	0.95	440	70.0	109.5	**0.75**
422	76.1	134.0	**0.75**	531	73.2	121.9	0.63
431/510	79.2	146.3	0.75/0.11	442/600	74.2	125.9	0.89/0.00

**Table 2:** Assessment of the XSA methods considered in this work concerning their suitability for analyzing various near-surface material conditions The order of the investigated states is identical to cases (*a*)–(*h*) in Fig. 8 ▸.

Near-surface material condition			XSA method		
σ_∥_	*a*_0_	*F*_∥_	MMWP	Constant-τ	Remarks
Constant	Constant	Constant	++	++	Evaluable without restrictions with both methods
*f*(*z*)	Constant	Constant	++	++	
Constant	*f*(*z*)	Constant	−	++	Evaluable without restrictions only with the constant-τ method
*f*(*z*)	*f*(*z*)	Constant	−	++	
Constant	Constant	*f*(*z*)	+	+	Evaluable with both methods if the analysis is confined to *hkl* near 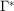
*f*(*z*)	Constant	*f*(*z*)	+	+	
Constant	*f*(*z*)	*f*(*z*)	−	+	Evaluable only with the constant-τ method if the analysis is confined to *hkl* near 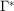
*f*(*z*)	*f*(*z*)	*f*(*z*)	−	+	

## Appendix A Direction-independent diffraction elastic constants

We express the DECs for both the Reuss (R) and the Voigt (V) in terms of single-crystal elastic moduli *s*_*ij*_. For the Reuss case (homogeneous stress in all crystallites) we obtain for materials possessing cubic symmetry (Möller & Martin, 1939 ▸)

with  and the orientation factor  = /. The DECs according to Voigt (homogeneous strain) do not depend on the orientation *hkl*:

The DECs according to the Eshelby–Kröner (K) model lie between the Voigt and Reuss limits. Since they also depend linearly on Γ^*hkl*^, they can be expressed by a weighting factor *r*^K^ using the Voigt and Reuss constants:

Fig. 18 ▸ reveals that the model functions intersect at a point 3.  is referred to as the model-independent orientation and can be obtained from the single-crystal elastic moduli by (Klaus & Genzel, 2019 ▸)

## Appendix B Direction-dependent diffraction elastic constants

We define direction-dependent (D) DECs according to the assumptions made for the surface layer  in Fig. 2 ▸ and write

The dashed framed areas in Fig. 19 ▸ show that the DECs vary less with the inclination angle ψ the closer the associated *hkl* are to the model-independent orientation . Note also that the conditions in this respect are more favorable for ferritic steel than for austenitic steel. One reason for this is that the elastic single-crystal anisotropy of ferrite (*A*_α_ = 2.5) is less pronounced than that for austenite (*A*_γ_ = 3.5). On the other hand, ferrite has more reflections (all those with 3Γ = 0.75) close to the model-independent orientation than austenite.

## Appendix C Strain in double-indexed lattice planes

Two lattice planes *h*_*i*_*k*_*i*_*l*_*i*_ and *h*_*j*_*k*_*j*_*l*_*j*_ in crystals with cubic symmetry whose Miller indices fulfill the condition  diffract at the same diffraction angle 2θ (AD case) or at the same energy *E*^*hkl*^ (ED case). However, since these lattice planes differ concerning their elastic behavior due to anisotropy, they will deform differently under the influence of a (residual) stress σ_∥_. In a polycrystalline material with a random crystallographic texture, there will therefore be a ‘shift as well as a splitting’ of the diffraction line. For experiments in ED diffraction mode assuming a biaxial stress state of rotational symmetry, the absolute shift of each diffraction line is given by

The relative displacement of the diffraction lines, referred to as *i* and *j* for brevity, is only dependent on *s*_0_, the weighting factor *r* and the difference between the orientation factors, Δ3Γ^(*i*, *j*)^ (see also Genzel *et al.*, 2023 ▸):

*hkl* in the above equation can stand for both *h*_*i*_*k*_*i*_*l*_*i*_ and *h*_*j*_*k*_*j*_*l*_*j*_. Fig. 20 ▸ shows the situation with the example of the diffraction line 333/511 for an austenitic steel. Due to the multiplicity factor (*H*^511^ = 24), the intensity of the 511 contribution to the total diffraction line is three times as high as the 333 contribution with a multiplicity factor *H*^333^ = 8. A prominent point for the rotationally symmetric in-plane stress state considered here is . At this point, the stress factors coincide independently of the grain interaction model (*cf*. Fig. 5 ▸), which means that the double-indexed diffraction lines do not split [Fig. 20 ▸(*b*)]. For smaller (larger) ψ angles, both line components shift towards smaller (larger) energies, whereby the shift of the 511 component (‘soft’ crystal direction) is always larger than that of the 333 component (‘hard’ direction).
